# A proteomic dataset of secreted proteins by three *Staphylococcus saprophyticus* strains

**DOI:** 10.1016/j.dib.2018.10.122

**Published:** 2018-10-27

**Authors:** Andrea Santana de Oliveira, Isabella Inês Rodrigues Rosa, Evandro Novaes, Lucas Silva de Oliveira, Lilian Cristiane Baeza, Clayton Luiz Borges, Lennart Marlinghaus, Célia Maria de Almeida Soares, Marcia Giambiagi-deMarval, Juliana Alves Parente-Rocha

**Affiliations:** a Laboratório de Biologia Molecular, Instituto de Ciências Biológicas, Universidade Federal de Goiás, Goiânia, Goiás, Brazil; b Escola de Agronomia, Universidade Federal de Goiás, Goiânia, Goiás, Brazil; c Centro de Ciências Médicas e Farmacêuticas, Universidade Estadual do Oeste do Paraná, Cascavel, Brazil; d Department of Medical Microbiology, Ruhr-University, Bochum, Germany; e Laboratório de Microbiologia Molecular, Instituto de Microbiologia Prof. Paulo de Góes, Universidade Federal do Rio de Janeiro, Rio de Janeiro, Brazil

## Abstract

This article presents a proteomic dataset generated from a comparative analysis of the exoproteome of *Staphylococcus saprophyticus,* ATCC 15305, 7108 and 9325 strains. The extract of secreted proteins were obtained after incubation of stationary phase cells in BHI medium. All samples were submitted to nano-ESI-UPLC-MS^E^, and the spectrum obtained was processed and analyzed by ProteinLynx Global Server (PLGS), Uniprot and Pedant databases, for identification, annotation and functional classification of proteins. Fold changes and protein relative abundances were properly reported. This report is related to the research article entitled “The exoproteome profiles of three *Staphylococcus saprophyticus* strains reveal diversity in protein secretion contents” (Oliveira et al., 2018). The proteomic data generated have been deposited to the ProteomeXchange Consortium, via the PRIDE partner repository, with a project number PXD008643, https://www.ebi.ac.uk/pride/archive/projects/PXD008643.

**Specifications table**

**Table:** 

Subject area	Molecular Microbiology
More specific subject area	Proteomic
Type of data	Table; figure
How data was acquired	Nanoscale LC separation of tryptic peptides was performed using a ACQUITY UPLC^®^ M-Class system (Waters Corporation, USA), and mass spectrometry analysis was performed on a Synapt G1 MS^TM^ (Waters, USA)
Data format	Filtered and Analyzed
Experimental factors	The secreted protein extract were obtained by TCA precipitation of culture supernatant obtained after incubation of *S.saprophyticus* cells in BHI medium. For protein identification, the samples were trypsin digested.
Experimental features	The secreted proteins were identified by nano-ESI-UPLC-MS^E^; the proteomic data was processed and analyzed by ProteinLynx Global Server (PLGS), Uniprot and Pedant databases. Statistical analysis were performed using R software.
Data source location	Goiânia, Goiás, Brazil.
Data accessibility	The analyzed data in excel format are available in this article, and raw data are accessible in *ProteomeXchange Consortium*[1]*, vi*a the PRIDE partner repository, under Project number PXD008643 [https://www.ebi.ac.uk/pride/archive/projects/PXD008643]
Related research article	Oliveira AS, Rosa IIR, Novaes E, Oliveira LS, Baeza LC, Borges CL, Marlinghaus L, Soares CMA, Giambiagi-deMarval M, A. P-RJ. The exoproteome profiles of three Staphylococcus saprophyticus strains reveal diversity in protein secretion contents. Microbiol Res. 2018 216: 85–96 [2]

**Value of the data**

• This dataset is an important step towards understanding the variability in *S. saprophyticus* exoproteome profiles. The data presented from *S.saprophyticus* exoproteomes pointed that the protein repertoire can be different among the strains showing metabolic flexibility can be involved in the ability to survive.
• The proteomic data present in this article might be significant in explaining differences among *S. saprophyticus* strains, since it will contribute in the identification of strain-specific virulence factors of *S. saprophyticus*.
• These comparative proteomic data will also contribute to support analysis to elucidate if differences in protein secretion can be related to *S. saprophyticus*׳s ability to cause infection.

## 1 Data

**Fig. 1:**
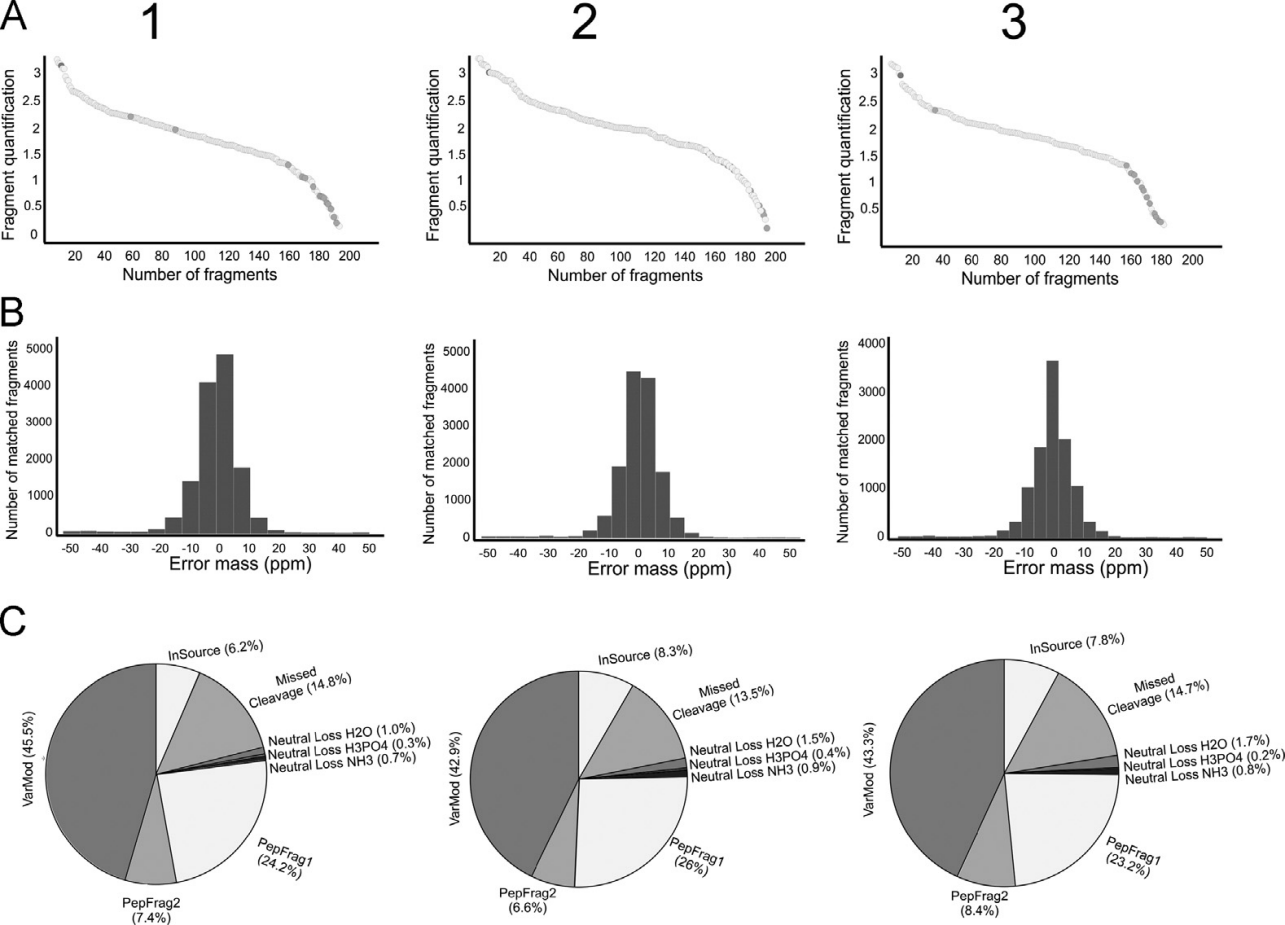
Data from mass spectrometry analysis. Columns 1 to 3 refers to data obtained from the samples ATCC 15305, 7108 and 9325, respectively. A: Detection dynamic range. Quantified fragments were sorted according to the fragment amount (Fmol) and plotted in the graphics. Standard protein was indicated by black circle. A protein with a low coefficient of variance between samples was used to normalize the expression data and allow comparisons. B: Mass error of the identified fragments. The number of identified fragments according to the error range (*x*-axis). C: Nano-ESI-UPLC-MS^**E**^ data quality analysis. PepFrag1 and PepFrag2 correspond to the peptides matches compared to the database by PLGS, VarMod corresponds to variable modifications, In Source corresponds to fragmentation that occurred in the ionization source, Missed Cleavage indicates the missed cleavage performed by trypsin and Neutral loss H2O and NH3 correspond to water and ammonia precursor losses.

This article presents a dataset generated from a comparative analysis of the exoproteome of three phenotypical different *Staphylococcus saprophyticus* strains (ATCC 15305, 7108 and 9325). The list of all proteins identified are provided in Supplementary Table 1, which the fold change of each protein׳s expression and protein relative abundance are shown. In this table, the amount of each protein quantified is shown in ngram. Detection dynamic range, mass error and proteomic quality data are shown in Fig. 1. This report presents data from the research article entitled “The exoproteome profiles of three *S. saprophyticus* strains reveal diversity in protein secretion contents”, where the differences encountered in the exoproteomes are described and validated [2].

## 2 Experimental design, materials and methods

The exoproteomes obtained from the three *S. saprophyticus* strains were submitted to nano-ESI-UPLC-MS^E^ for total protein identification, as described below. The data showed in this article were generated by a comparative proteomic analysis performed using statistical tools from R software.

## 3 Protein identification by nano-ESI-UPLC-MS^E^

The extract of secreted proteins from three *S. saprophyticus* strains were obtained from the culture supernatant in the stationary phase. The protein precipitation was performed by addiction of Trichloroacetic acid (TCA) (Sigma-Aldrich, St. Louis, MO, USA) at a final concentration of 10% (w/v). After obtainment of a secreted protein extract of the three *S. saprophyticus* strains, a total of 500 µg of each protein sample was used for enzymatic digestion by trypsin, that were processed according as described [2]. ACQUITY UPLC^®^ M-Class system (Waters Corporation, USA) was used for nanoscale LC separation of digested peptides, and Synapt G1 MS^TM^ (Waters, USA) used for analysis of mass spectrometry, as previous described [3]. All samples were analyzed from three experimental replicates. The mass error tolerance for each peptide identified was under 50 ppm. The protein identification criteria also included the detection of at least 2 fragment ions per peptide, 5 fragments per protein and the determination of at least 1 peptide per protein, as showed in spectrometry data analysis in Fig. 1.

## 4 Processing and proteomic data analysis

The data from mass spectrometry were processed by ProteinLynx Global Server (PLGS) version 3.0.2 (Waters, Manchester, UK), and Uniprot Database (http://www.uniprot.proteomes/), by searching proteins sequences from *S. saprophyticus*. The annotation and functional classification of proteins identified was made by Uniprot (http://www.uniprot.org), and Pedant in the MIPS (http://mips.helmholtz-muenchen.de/funcatDB/) database. The NCBI database (https://www.ncbi.nlm.nih.gov) was used for annotation of proteins that were not characterized.

In order to measure of protein expression, the *n*-gram of each protein identified in *S. saprophyticus* was used. R software was used to combine the generated data into a matrix, where rows indicated the proteins, and columns the samples (strains × replicates). Proteins were described as differentially expressed using a threshold of 0.05 false discovery. The fold change of each protein׳s expression was showed in logarithm scale and was calculated by comparison of the quantification in strains 7108 and 9325 with the level in the ATCC 15305 strain. The protein abundance was calculated by analyzing the protein data from the technical replicate of the strains (Supplementary Table 1).
