# Supplementary material for: A proteomic dataset of secreted proteins by three Staphylococcus saprophyticus strains
Source: Data Brief. 2018 Oct 27;21:1472–6. doi: 10.1016/j.dib.2018.10.122 (PMC6234272; doi:10.1016/j.dib.2018.10.122)
Supplement: Supplementary file 1 — Supplementary material [file mmc1.pdf]

AUTHOR DECLARATION

The authors confirm that there are no known conflicts of interest associated with this publication and there has been no significant financial support for this work that could have influenced its outcome. The authors understand that the Corresponding Author is the sole contact for the Editorial process (including Editorial Manager and direct communications with the office). The corresponding author is responsible for communicating with the other authors about progress, submissions of revisions and final approval of proofs. The authors e-mails were included in the submission form and they are aware of the manuscript submission.

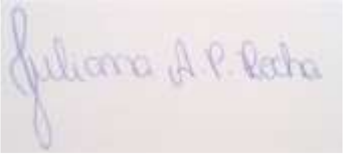

Juliana Alves Parente Rocha
juparente@gmail.com
Phone: 55 62 3521 1110
Corresponding author
