# Supplementary material for: A proteomic dataset of secreted proteins by three Staphylococcus saprophyticus strains
Source: Data Brief. 2018 Oct 27;21:1472–6. doi: 10.1016/j.dib.2018.10.122 (PMC6234272; doi:10.1016/j.dib.2018.10.122)
Supplement: Supplementary file 2 — Supplementary material [file mmc2.docx]

**Table 1 –** List of proteins identified in the exoproteome of three *S. saprophyticus* strains.

| **Accession number^1^** | **Protein description** | **Log FC^2^** | | **t-statistic^3^** | | **p-value^4^** | | **ngram^5^** | | | **% in the exoproteome^6^** | | | | **MW (Da)** |
| --- | --- | --- | --- | --- | --- | --- | --- | --- | --- | --- | --- | --- | --- | --- | --- |
|  |  | **7108** | **9325** | **7108** | **9325** | **7108** | **9325** | **ATCC 15305** | **7108** | **9325** | **ATCC 15305** | **7108** | **9325** | |  |
| **Glycolysis** | |  |  |  |  |  |  |  |  |  |  |  |  | |  |
| ENO_STAS1 | Enolase | 0.64 | 0.54 | 3.07 | 2.59 | 0.031 | 0.092 | 13.17 | 7.44 | 8.25 | 1.16 | 0.65 | 1.13 | | 47132.05 |
| TPIS_STAS1 | Triosephosphate isomerase | 0.37 | -0.41 | 2.42 | -2.67 | 0.074 | 0.086 | 4.60 | 3.74 | 5.43 | 0.40 | 0.32 | 0.74 | | 27495.01 |
| Q49VZ9_STAS1 | Glyceraldehyde-3-phosphate dehydrogenase | 1.26 | -0.69 | 8.69 | -4.75 | ≤ 0.001 | 0.009 | 16.23 | 6.18 | 21.16 | 1.43 | 0.54 | 2.89 | | 36316.82 |
| PGK_STAS1 | Phosphoglycerate kinase | 1.07 | -0.14 | 5.72 | -0.72 | 0.002 | 0.633 | 4.42 | 2.17 | 4.45 | 0.39 | 0.19 | 0.61 | | 42451.31 |
| Q4A0Q6_STAS1 | Fructose-bisphosphate aldolase class I | 0.19 | 0.34 | 0.90 | 1.62 | 0.481 | 0.291 | 9.99 | 7.17 | 6.19 | 0.88 | 0.62 | 0.85 | | 33198.59 |
| Q49Z72_STAS1 | Fructose-bisphosphate aldolase class I | -0.12 | 0.34 | -0.57 | 1.63 | 0.667 | 0.291 | 2.80 | 3.04 | 1.92 | 0.25 | 0.26 | 0.26 | | 30797.83 |
| **Piruvate dehydrogenase complex** | |  |  |  |  |  |  |  |  |  |  |  |  | |  |
| Q49WM1_STAS1 | Dihydrolipoyl dehydrogenase | -2.02 | -0.12 | -7.39 | -0.46 | ≤ 0.001 | 0.805 | 11.07 | 33.47 | 9.44 | 0.97 | 2.91 | 1.29 | | 49681.55 |
| Q49WL9 | Pyruvate dehydrogenase E1 | 0.43 | -0.02 | 1.64 | -0.06 | 0.200 | 1.000 | 1.51 | ** | 1.30 | 0.13 | ** | 0.18 | | 35237.96 |
| Q49WM0 | Dihydrolipoamide S-acetyltransferase component of pyruvate dehydrogenase complex E2 | -0.64 | 0.56 | -1.04 | 0.92 | 0.404 | 0.547 | ** | 3.89 | ** | ** | 0.34 | ** | | 46645.27 |
| **Lactic fermentation** | |  |  |  |  |  |  |  |  |  |  |  |  | |  |
| LDH | L-lactate dehydrogenase | 2.15 | 0.00 | 13.86 | 0.03 | ≤ 0.001 | 1.000 | 19.02 | 3.84 | 15.24 | 1.67 | 0.33 | 2.08 | | 34580.42 |
| **Pentose phosphate pathway** | |  |  |  |  |  |  |  |  |  |  |  |  | |  |
| Q49YL0 | Transaldolase | -0.98 | -0.15 | -8.27 | -1.29 | ≤ 0.001 | 0.418 | 9.90 | 15.35 | 8.92 | 0.87 | 1.33 | 1.22 | | 25662.13 |
| Q49XD6 | Transketolase | 0.54 | 0.05 | 3.69 | 0.37 | 0.014 | 0.865 | 18.19 | 10.55 | 13.31 | 1.60 | 0.92 | 1.82 | | 72183.09 |
| Q49XV6 | 6-phosphogluconate dehydrogenase decarboxylating | -0.66 | 0.62 | -5.16 | 4.83 | 0.003 | 0.009 | 8.01 | 11.51 | 4.61 | 0.70 | 1.00 | 0.63 | | 52080.67 |
| Q49XV2 | Glucose-6-phosphate 1-dehydrogenase | -3.44 | 0.00 | -35.70 | 0.00 | ≤ 0.001 | 1.000 | ** | 10.21 | ** | ** | 0.89 | ** | | 56977.31 |
| Q49X03 | Ribulose-phosphate 3-epimerase | 0.57 | 0.00 | 2.15 | 0.00 | 0.103 | 1.000 | ** | 0.86 | ** | ** | 0.07 | ** | | 23554.99 |
| **C-compounds metabolism** | |  |  |  |  |  |  |  |  |  |  |  |  | |  |
| GPMA | 2,3-bisphosphoglycerate-dependent phosphoglycerate mutase | -1.70 | -0.11 | -9.59 | -0.64 | ≤ 0.001 | 0.680 | 4.60 | 13.53 | 4.66 | 0.40 | 1.18 | 0.64 | | 26463.89 |
| GPMI | 2,3-bisphosphoglycerate-independent phosphoglycerate mutase | -2.08 | 0.80 | -5.83 | 2.24 | 0.002 | 0.141 | 2.39 | 7.21 | ** | 0.21 | 0.63 | ** | | 56400.06 |
| Q49ZH5 | Glucose-1-dehydrogenase | 0.06 | -0.84 | 0.13 | -1.76 | 0.947 | 0.257 | ** | 1.00 | 1.44 | ** | 0.09 | 0.20 | | 28460.54 |
| HPS1 | 3-hexulose-6-phosphate synthase 1 | -2.59 | -0.59 | -5.28 | -1.20 | 0.003 | 0.433 | ** | 4.38 | ** | ** | 0.38 | ** | | 22352.55 |
| Q49VE1 | Phosphotransacetylase | -2.04 | 0.13 | -14.25 | 0.90 | ≤ 0.001 | 0.550 | ** | 4.54 | ** | ** | 0.39 | ** | | 35301.06 |
| **Fatty acid metabolism** | |  |  |  |  |  |  |  |  |  |  |  |  | |  |
| ACP | Acyl carrier protein | -1.53 | -0.05 | -4.97 | -0.16 | 0.004 | 1.000 | 1.29 | 3.82 | 1.41 | 0.11 | 0.33 | 0.19 | | 8563.48 |
| Q49WB7 | 3-oxoacyl-[acyl-carrier-protein] synthase | -1.38 | -0.92 | -4.29 | -2.85 | 0.007 | 0.068 | ** | 3.57 | 2.18 | ** | 0.31 | 0.30 | | 43704.99 |
| **Isoprenoid metabolism** | |  |  |  |  |  |  |  |  |  |  |  |  | |  |
| ISPD | 2-c-methyl-d-erythritol 4-phosphate cytidylyltransferase | -1.66 | -1.37 | -4.35 | -3.60 | 0.007 | 0.028 | 0.60 | 1.82 | 1.25 | 0.05 | 0.16 | 0.17 | | 26420.31 |
| **ATP synthesis** | |  |  |  |  |  |  |  |  |  |  |  |  | |  |
| ATPA | ATP synthase subunit alpha | -0.81 | -0.29 | -5.68 | -2.02 | 0.002 | 0.184 | 5.80 | 9.67 | 6.27 | 0.51 | 0.84 | 0.86 | | 54577.29 |
| ATPB | ATP synthase subunit beta | -1.46 | 0.43 | -2.33 | 0.69 | 0.080 | 0.655 | 3.20 | 6.14 | ** | 0.28 | 0.53 | ** | | 51220.08 |
| Y1834 | NADH dehydrogenase-like protein | 0.00 | -2.26 | 0.00 | -15.83 | 1.000 | ≤ 0.001 | ** | ** | 4.77 | ** | ** | 0.65 | | 44455.60 |
| **Nucleotide and nucleoside metabolism** | |  |  |  |  |  |  |  |  |  |  |  |  | |  |
| Q49Z85 | Purine nucleoside phosphorylase DeoD | -1.68 | -0.19 | -5.96 | -0.66 | 0.001 | 0.664 | 1.33 | 4.09 | 1.54 | 0.12 | 0.36 | 0.21 | | 26197.55 |
| Q49XS7 | Bacterial nucleoid DNA-binding protein | -1.66 | -0.40 | -14.07 | -3.39 | ≤ 0.001 | 0.036 | 3.33 | 9.54 | 4.03 | 0.29 | 0.83 | 0.55 | | 9641.08 |
| NDK | Nucleoside diphosphate kinase | -0.09 | 0.03 | -0.30 | 0.10 | 0.827 | 1.000 | 1.03 | 1.29 | ** | 0.09 | 0.11 | ** | |  |
| **Amino acids metabolism** | |  |  |  |  |  |  |  |  |  |  |  |  | |  |
| Q49XA2 | Glutamine synthetase | -0.84 | 1.26 | -5.23 | 7.84 | 0.003 | 0.001 | 2.54 | 4.39 | ** | 0.22 | 0.38 | ** | | 51161.03 |
| GCSH | Glycine cleavage system H protein | -1.04 | 0.10 | -4.40 | 0.42 | 0.006 | 0.833 | ** | 2.21 | 0.78 | ** | 0.19 | 0.11 | | 14101.51 |
| DHA | Alanine dehydrogenase | 1.94 | 1.94 | 19.18 | 19.18 | ≤ 0.001 | ≤ 0.001 | 4.16 | ** | ** | 0.37 | ** | ** | | 39959.78 |
| Q49W97 | Glutamate dehydrogenase | 0.50 | 0.50 | 4.28 | 4.28 | 0.007 | 0.013 | 1.41 | ** | ** | 0.12 | ** | ** | | 45919.08 |
| **DNA repair** | |  |  |  |  |  |  |  |  |  |  |  |  | |  |
| END4 | Probable endonuclease 4 | 0.22 | 0.33 | 0.86 | 1.27 | 0.501 | 0.424 | ** | 1.02 | ** | ** | 0.09 | ** | | 33313.73 |
| **Cell signaling** | |  |  |  |  |  |  |  |  |  |  |  |  | |  |
| Q49ZE7 | Adenylate kinase | -1.29 | 0.08 | -4.21 | 0.26 | 0.008 | 0.936 | 1.33 | 3.18 | 1.26 | 0.12 | 0.28 | 0.17 | | 24388.81 |
| PPAC | Manganese-dependent inorganic pyrophosphatase | 0.36 | 0.36 | 1.66 | 1.67 | 0.195 | 0.285 | 1.46 | 1.14 | ** | 0.13 | 0.10 | ** | | 34007.36 |
| **Cell division** | |  |  |  |  |  |  |  |  |  |  |  |  | |  |
| Q49Y87 | Putative cell-shape determining protein | -1.16 | 0.00 | -13.72 | 0.00 | ≤ 0.001 | 1.000 | ** | 2.45 | ** | ** | 0.21 | ** | | 31067.98 |
| **Cell wall synthesis** | |  |  |  |  |  |  |  |  |  |  |  |  | |  |
| ISAA | Probable transglycosylase IsaA | 0.11 | -0.36 | 0.23 | -0.75 | 0.874 | 0.623 | 41.50 | 29.66 | 42.22 | 3.65 | 2.58 | 5.77 | | 25511.41 |
| SCED1 | Probable transglycosylase sceD 1 | 1.40 | -0.34 | 10.32 | -2.50 | ≤ 0.001 | 0.099 | 11.91 | 3.60 | 12.79 | 1.05 | 0.31 | 1.75 | | 24928.32 |
| SCED2 | Probable transglycosylase sceD 2 | 0.49 | -0.97 | 1.52 | -3.00 | 0.228 | 0.059 | 33.89 | 16.98 | 40.78 | 2.98 | 1.47 | 5.58 | | 25828.87 |
| Q49WH3 | Bifunctional autolysin | 1.35 | 0.00 | 14.55 | 0.00 | ≤ 0.001 | 1.000 | 315.99 | 114.24 | 220.15 | 27.79 | 9.92 | 30.10 | | 159412.25 |
| SLE1 | N-acetylmuramoyl-L-alanine amidase sle1 | 0.77 | -0.12 | 1.75 | -0.27 | 0.175 | 0.928 | 2.27 | ** | 2.23 | 0.20 | ** | 0.31 | | 34548.44 |
| LTAS | Lipoteichoic acid synthase | 5.34 | 0.99 | 32.40 | 6.00 | ≤ 0.001 | 0.003 | 45.50 | ** | 17.77 | 4.00 | ** | 2.43 | | 74912.43 |
| Q49XP8 | Putative extracellular amidase | 1.03 | 1.03 | 9.19 | 9.19 | ≤ 0.001 | ≤ 0.001 | 1.96 | ** | ** | 0.17 | ** | ** | | 29408.27 |
| Q49UH9 | Mannosyl-glycoprotein endo-beta-N-acetylglucosamidase-like domain | 4.87 | 3.71 | 5.79 | 4.40 | 0.002 | 0.013 | 32.49 | ** | ** | 2.86 | ** | ** | | 78923.11 |
| **Transcription** | |  |  |  |  |  |  |  |  |  |  |  |  | |  |
| Q49ZN1 | Putative transcriptional regulator | 0.00 | -1.42 | 0.00 | -15.10 | 1.000 | ≤ 0.001 | ** | ** | 2.41 | ** | ** | 0.33 | | 34866.19 |
| Q49WH6 | Transcriptional regulator | 1.58 | 2.09 | 3.76 | 4.98 | 0.013 | 0.007 | 5.51 | 2.49 | ** | 0.48 | 0.22 | ** | | 48680.80 |
| RPOA | DNA-directed RNA polymerase subunit alpha | 0.71 | 1.10 | 1.57 | 2.41 | 0.215 | 0.112 | 3.26 | 1.61 | ** | 0.29 | 0.14 | ** | | 35036.87 |
| CODY | GTP-sensing transcriptional pleiotropic repressor | -1.01 | -0.59 | -1.93 | -1.13 | 0.137 | 0.456 | 0.39 | ** | ** | 0.03 | ** | ** | | 28542.84 |
| GREA | Transcription elongation factor GreA | -0.68 | 0.42 | -1.91 | 1.18 | 0.139 | 0.442 | ** | 1.74 | ** | ** | 0.15 | ** | | 17835.03 |
| RPOE | Probable DNA-directed RNA polymerase subunit | -0.67 | 0.00 | -3.81 | 0.00 | 0.012 | 1.000 | ** | 1.74 | ** | ** | 0.15 | ** | | 21474.56 |
| Q49Z44 | Single-stranded DNA-binding protein | 0.75 | -0.33 | 1.12 | -0.49 | 0.371 | 0.781 | ** | ** | 2.80 | ** | ** | 0.38 | | 16230.55 |
| **Protein synthesis** | |  |  |  |  |  |  |  |  |  |  |  |  | |  |
| RS5 | 30S ribosomal protein S5 | 0.00 | -0.07 | 0.00 | -0.73 | 1.000 | 0.633 | ** | ** | 0.98 | ** | ** | 0.13 | | 17582.44 |
| RS6 | 30S ribosomal protein S6 | -1.14 | -0.24 | -4.92 | -1.03 | 0.004 | 0.500 | ** | 2.56 | 1.08 | ** | 0.22 | 0.15 | | 11933.42 |
| RS7 | 30S ribosomal protein S7 | 0.33 | -1.12 | 2.18 | -7.35 | 0.099 | 0.001 | 1.22 | ** | 2.46 | 0.11 | ** | 0.34 | | 17825.59 |
| RS8 | 30S ribosomal protein S8 | -0.01 | 0.29 | -0.06 | 1.60 | 0.988 | 0.294 | 0.92 | ** | 0.76 | 0.08 | ** | 0.10 | | 14806.24 |
| RS9 | 30S ribosomal protein S9 | 0.43 | 0.77 | 1.27 | 2.26 | 0.319 | 0.140 | 1.68 | ** | ** | 0.15 | ** | ** | | 14554.66 |
| RS10 | 30S ribosomal protein S10 | 1.25 | 0.05 | 2.58 | 0.10 | 0.058 | 1.000 | 1.25 | 0.45 | 1.12 | 0.11 | 0.04 | 0.15 | | 11590.48 |
| RS16 | 30S ribosomal protein S16 | -0.79 | 0.31 | -3.09 | 1.23 | 0.031 | 0.426 | 1.01 | 2.06 | 0.72 | 0.09 | 0.18 | 0.10 | | 9973.45 |
| RS19 | 30S ribosomal protein S19 | -0.50 | 0.00 | -3.90 | 0.00 | 0.011 | 1.000 | ** | 1.57 | ** | ** | 0.14 | ** | | 10485.06 |
| RL2 | 50S ribosomal protein L2 | -0.70 | -1.06 | -1.44 | -2.20 | 0.252 | 0.146 | ** | 1.82 | 2.30 | ** | 0.16 | 0.31 | | 30415.32 |
| RL5 | 50S ribosomal protein L5 | -0.08 | -1.09 | -0.59 | -7.99 | 0.652 | 0.001 | 0.99 | ** | 1.82 | 0.09 | ** | 0.25 | | 20215.43 |
| RL6 | 50S ribosomal protein L6 | 0.10 | 0.53 | 0.35 | 1.80 | 0.813 | 0.249 | 1.74 | 1.97 | 1.13 | 0.15 | 0.17 | 0.15 | | 19569.29 |
| RL7 | 50S ribosomal protein L7/L12 | -1.30 | 0.16 | -11.18 | 1.41 | ≤ 0.001 | 0.373 | 5.34 | 12.15 | 4.38 | 0.47 | 1.05 | 0.60 | | 12661.46 |
| RL9 | 50S ribosomal protein L9 | -0.18 | 0.37 | -0.59 | 1.26 | 0.652 | 0.425 | ** | 1.27 | ** | ** | 0.11 | ** | | 16464.89 |
| RL11 | 50S ribosomal protein L11 | -0.79 | 0.04 | -5.26 | 0.27 | 0.003 | 0.928 | 1.41 | 2.83 | 1.36 | 0.12 | 0.25 | 0.19 | | 14974.38 |
| RL15 | 50S ribosomal protein L15 | -0.57 | 0.38 | -1.17 | 0.78 | 0.351 | 0.616 | 2.47 | 3.77 | 1.89 | 0.22 | 0.33 | 0.26 | | 15469.73 |
| RL17 | 50S ribosomal protein L17 | 0.19 | 0.55 | 0.44 | 1.25 | 0.751 | 0.426 | ** | 0.92 | ** | ** | 0.08 | ** | | 13702.77 |
| RL20 | 50S ribosomal protein L20 | 0.78 | 0.54 | 1.54 | 1.07 | 0.223 | 0.483 | ** | 0.49 | ** | ** | 0.04 | ** | | 13720.24 |
| RL21 | 50S ribosomal protein L21 | 0.84 | 0.22 | 3.25 | 0.87 | 0.025 | 0.554 | 1.26 | 0.79 | ** | 0.11 | 0.07 | ** | | 11290.01 |
| RL22 | 50S ribosomal protein L22 | 0.66 | -0.07 | 2.34 | -0.25 | 0.080 | 0.936 | ** | 0.73 | ** | ** | 0.06 | ** | | 12806.95 |
| RL24 | 50S ribosomal protein L24 | -0.11 | 0.19 | -0.65 | 1.13 | 0.623 | 0.456 | ** | 1.24 | ** | ** | 0.11 | ** | | 11544.34 |
| RL25 | 50S ribosomal protein L25 | -0.34 | 0.73 | -1.87 | 4.07 | 0.147 | 0.016 | 4.33 | 5.49 | 2.39 | 0.38 | 0.48 | 0.33 | | 24124.88 |
| RL27 | 50S ribosomal protein L27 | -0.50 | 0.00 | -4.46 | 0.00 | 0.006 | 1.000 | ** | 1.54 | ** | ** | 0.13 | ** | | 10286.72 |
| RL29 | 50S ribosomal protein L29 | -0.97 | -0.25 | -3.81 | -0.96 | 0.012 | 0.532 | 1.25 | 2.68 | 1.43 | 0.11 | 0.23 | 0.20 | | 8060.23 |
| RL31B | 50S ribosomal protein L31 type B | 0.14 | 0.48 | 0.38 | 1.29 | 0.791 | 0.418 | ** | 0.99 | ** | ** | 0.09 | ** | | 9886.89 |
| RL32 | 50S ribosomal protein L32 | -0.99 | -0.43 | -2.81 | -1.23 | 0.045 | 0.426 | 0.72 | 1.79 | ** | 0.06 | 0.16 | ** | | 6756.76 |
| RL35 | 50S ribosomal protein L35 | -0.51 | -0.56 | -2.96 | -3.29 | 0.036 | 0.040 | 0.63 | 1.12 | ** | 0.06 | 0.10 | ** | | 7614.23 |
| EFTS | Elongation factor Ts | -0.55 | 0.65 | -2.36 | 2.77 | 0.080 | 0.075 | 14.13 | 16.56 | 7.54 | 1.24 | 1.44 | 1.03 | | 32386.74 |
| EFTU | Elongation factor Tu | 1.37 | 0.30 | 7.42 | 1.63 | ≤ 0.001 | 0.291 | 8.22 | 3.00 | 5.42 | 0.72 | 0.26 | 0.74 | | 43278.79 |
| SYE | Glutamate--tRNA ligase | -1.50 | -1.32 | -3.32 | -2.91 | 0.023 | 0.064 | 2.33 | 5.13 | 4.58 | 0.20 | 0.45 | 0.63 | | 56443.81 |
| Q49WL6 | Peptide deformylase 2 | -0.83 | -0.48 | -1.60 | -0.92 | 0.210 | 0.547 | ** | 1.53 | ** | ** | 0.13 | ** | | 20508.53 |
| RRF | Ribosome-recycling factor | -1.22 | 0.15 | -9.10 | 1.12 | ≤ 0.001 | 0.461 | 2.83 | 6.33 | 2.11 | 0.25 | 0.55 | 0.29 | | 20363.86 |
| TIG | Trigger factor | -0.51 | 0.12 | -3.20 | 0.77 | 0.027 | 0.618 | 3.70 | 5.37 | 2.97 | 0.33 | 0.47 | 0.41 | | 48878.99 |
| **Protein folding, degradation and modification** | |  |  |  |  |  |  |  |  |  |  |  |  | |  |
| Q4A0H6 | Glutamyl endopeptidase | 1.05 | -0.64 | 7.65 | -4.70 | ≤ 0.001 | 0.009 | 3.10 | 3.16 | 2.99 | 0.27 | 0.27 | 0.41 | | 28093.14 |
| CLPP | ATP-dependent Clp protease proteolytic subunit | -0.85 | 0.09 | -8.23 | 0.90 | ≤ 0.001 | 0.550 | 4.35 | 8.39 | 2.57 | 0.38 | 0.73 | 0.35 | | 21268.23 |
| Q49WC9 | Oligopeptidase F | -2.39 | -1.09 | -3.17 | -1.45 | 0.027 | 0.360 | 6.80 | 55.98 | 10.67 | 0.60 | 4.86 | 1.46 | | 69940.00 |
| Y1059 | Xaa-Pro aminopeptidase | -1.66 | -0.83 | -4.40 | -2.19 | 0.006 | 0.146 | ** | 4.96 | 0.96 | ** | 0.43 | 0.13 | | 39443.46 |
| Q49YS4 | Leucyl aminopeptidase | -1.98 | 0.00 | -11.02 | 0.00 | ≤ 0.001 | 1.000 | ** | 5.63 | ** | ** | 0.49 | ** | | 46598.81 |
| Q49YT3 | Methionine aminopeptidase | 0.02 | 0.00 | 0.09 | 0.00 | 0.976 | 0.433 | ** | 2.60 | ** | ** | 0.23 | ** | | 27427.21 |
| Q49XX4 | Hyperthermophile prolidase | -2.01 | 0.00 | -14.53 | 0.00 | ≤ 0.001 | 1.000 | ** | 6.17 | ** | ** | 0.54 | ** | | 38787.09 |
| PPI1 | Putative peptidyl-prolyl cis-trans isomerase | -0.38 | 0.00 | -1.25 | 0.00 | 0.324 | 1.000 | ** | 2.78 | ** | ** | 0.24 | ** | | 21683.33 |
| Q49ZX5 | Putative small heat shock protein | 0.86 | 0.00 | 1.93 | 0.00 | 0.137 | 1.000 | ** | 0.72 | ** | ** | 0.06 | ** | | 16595.24 |
| CH10 | 10 kDa chaperonin | -1.89 | 0.82 | -7.07 | 3.06 | ≤ 0.001 | 0.055 | 1.78 | 6.76 | 0.91 | 0.16 | 0.59 | 0.12 | | 10331.69 |
| CH60 | 60 kDa chaperonin | 0.48 | 1.27 | 2.72 | 7.12 | 0.048 | 0.001 | 45.33 | 30.29 | 15.89 | 3.99 | 2.63 | 2.17 | | 57757.50 |
| GRPE | Protein grpE | -1.12 | 0.84 | -4.93 | 3.70 | 0.004 | 0.025 | 1.77 | 3.82 | ** | 0.16 | 0.33 | ** | | 23587.59 |
| DNAK | Chaperone protein DnaK | 0.22 | 3.15 | 0.30 | 4.33 | 0.827 | 0.013 | 21.10 | 14.27 | ** | 1.86 | 1.24 | ** | | 66642.26 |
| **Nitrogen metabolism** | |  |  |  |  |  |  |  |  |  |  |  |  | |  |
| UREG | Urease accessory protein UreG | 1.41 | 1.47 | 9.41 | 9.81 | ≤ 0.001 | ≤ 0.001 | 2.82 | ** | ** | 0.25 | ** | ** | | 22427.58 |
| URE1 | Urease subunit alpha | -2.02 | 1.00 | -2.69 | 1.33 | 0.050 | 0.408 | ** | 8.91 | ** | ** | 0.77 | ** | | 61942.07 |
| **Iron metabolism** | |  |  |  |  |  |  |  |  |  | 0.00 | 0.00 | 0.00 | |  |
| FTN | Ferritin | -1.45 | 0.52 | -10.31 | 3.68 | ≤ 0.001 | 0.025 | 1.85 | 5.29 | 1.30 | 0.16 | 0.46 | 0.18 | | 19654.57 |
| Q49WI6 | Putative flavohemoprotein | 1.44 | -0.87 | 2.72 | -1.65 | 0.048 | 0.290 | 4.62 | ** | 4.70 | 0.41 | ** | 0.64 | | 42730.87 |
| **Transport** | |  |  |  |  |  |  |  |  |  |  |  |  | |  |
| Q49ZK8 | ABC-type cobalamin Fe3+siderophore transport system | -0.80 | -0.75 | -2.78 | -2.60 | 0.046 | 0.092 | 1.49 | 2.43 | 2.06 | 0.13 | 0.21 | 0.28 | | 34623.48 |
| Q4A0L3 | ABC-type amino acid transport system permease | -0.15 | 0.49 | -1.18 | 3.76 | 0.351 | 0.024 | ** | 1.25 | 0.67 | ** | 0.11 | 0.09 | | 29575.63 |
| Q49XG0 | Ribose ABC transporter | 0.97 | 0.97 | 9.84 | 9.84 | ≤ 0.001 | ≤ 0.001 | 2.04 | ** | ** | 0.18 | ** | ** | | 32911.70 |
| Q49XC5 | Putative phosphate phosphonate-binding protein ABC | 2.34 | 2.34 | 14.19 | 14.19 | ≤ 0.001 | ≤ 0.001 | 5.58 | ** | ** | 0.49 | ** | ** | | 35495.01 |
| Q49XN3 | PTS system glucose-specific IIA component | -0.09 | -0.09 | -0.52 | -0.52 | 0.699 | 0.768 | 0.98 | ** | ** | 0.09 | ** | ** | | 17808.32 |
| Q49W50 | Lipoprotein NlpA family | -1.01 | 0.00 | -7.67 | 0.00 | ≤ 0.001 | 1.000 | ** | 2.26 | ** | ** | 0.20 | ** | | 30084.66 |
| **Cofators and vitamins metabolism** | |  |  |  |  |  |  |  |  |  |  |  |  | |  |
| PDXS | Pyridoxal biosynthesis lyase PdxS | 0.28 | -1.50 | 2.13 | -11.25 | 0.104 | ≤ 0.001 | 1.31 | ** | 3.17 | 0.12 | ** | 0.43 | | 32020.80 |
| **Stress response** | |  |  |  |  |  |  |  |  |  |  |  |  | |  |
| AHPC | Alkyl hydroperoxide reductase subunit C | -1.88 | -0.94 | -15.42 | -7.71 | ≤ 0.001 | 0.001 | 5.85 | 18.93 | 10.62 | 0.51 | 1.64 | 1.45 | | 21207.54 |
| CATA | Catalase | 0.24 | 1.02 | 2.30 | 9.80 | 0.084 | ≤ 0.001 | 13.76 | 9.30 | 5.52 | 1.21 | 0.81 | 0.75 | | 57340.68 |
| TPX | Probable thiol peroxidase | -0.32 | 0.14 | -1.59 | 0.68 | 0.211 | 0.657 | 0.95 | 1.27 | 0.65 | 0.08 | 0.11 | 0.09 | | 18370.57 |
| Q49Z86 | Starvation-inducible DNA-binding protein | -0.03 | 0.63 | -0.33 | 6.48 | 0.817 | 0.002 | 7.90 | 7.29 | 4.46 | 0.69 | 0.63 | 0.61 | | 16981.05 |
| SODM | Superoxide dismutase [Mn/Fe] | -1.51 | 0.09 | -15.24 | 0.88 | ≤ 0.001 | 0.554 | 5.63 | 14.17 | 4.76 | 0.50 | 1.23 | 0.65 | | 22593.17 |
| THIO | Thioredoxin | -1.51 | -0.28 | -6.90 | -1.30 | 0.001 | 0.418 | 1.45 | 4.18 | 1.60 | 0.13 | 0.36 | 0.22 | | 11552.19 |
| Q49W43 | Thioredoxin | -0.71 | -0.28 | -2.75 | -1.10 | 0.048 | 0.469 | ** | 1.50 | ** | ** | 0.13 | ** | | 12225.69 |
| Y1056 | Putative universal stress protein | 1.55 | 0.88 | 4.67 | 2.64 | 0.005 | 0.088 | 3.05 | ** | 2.02 | 0.27 | ** | 0.28 | | 18623.92 |
| QOX2 | Probable quinol oxidase subunit 2 | 2.53 | 2.53 | 13.32 | 13.32 | ≤ 0.001 | ≤ 0.001 | 6.14 | ** | ** | 0.54 | ** | ** | | 42722.56 |
| Q49WV6 | Putative antibacterial protein | -0.66 | -0.66 | -4.35 | -4.35 | 0.007 | 0.013 | 0.58 | ** | ** | 0.05 | ** | ** | | 4439.15 |
| **Virulence factor** | |  |  |  |  |  |  |  |  |  |  |  |  | |  |
| UAFA | Uro-adherence factor A | -1.41 | 0.00 | -14.66 | 0.00 | ≤ 0.001 | ≤ 0.001 | 114.91 | 259.04 | ** | 10.11 | 22.49 | ** | | 245869.65 |
| Q49VK7 | Uro-adherence factor A | 0.00 | -1.75 | 0.00 | -2.88 | 0.048 | 0.066 | 5.80 | ** | 10.78 | 0.51 | ** | 1.47 | | 28313.55 |
| Q49ZL8 | Putative secretory antigen | 0.00 | -0.26 | 0.00 | -1.67 | ≤ 0.001 | 0.285 | 8.88 | ** | 9.33 | 0.78 | ** | 1.28 | | 24976.15 |
| Q49ZM2 | Putative secretory antigen | 0.00 | -1.44 | 0.00 | -1.81 | 0.328 | 0.248 | 3.52 | ** | 9.96 | 0.31 | ** | 1.36 | | 15973.08 |
| Q4A0W2 | Immunodominant antigen | 0.12 | 1.08 | 0.61 | 5.50 | 0.652 | 0.004 | 3.28 | 2.99 | 2.61 | 0.29 | 0.26 | 0.36 | | 5728.41 |
| **Uncharacterized proteins** | |  |  |  |  |  |  |  |  |  |  |  |  | |  |
| Q49ZY4 | Uncharacterized protein | -1.00 | -1.10 | -3.61 | -3.98 | 0.015 | 0.018 | 0.92 | 1.99 | 1.76 | 0.08 | 0.17 | 0.24 | | 19174.36 |
| Q49VC9 | Uncharacterized protein | 0.62 | -0.66 | 4.04 | -4.26 | 0.009 | 0.013 | 24.65 | 13.08 | 27.10 | 2.17 | 1.14 | 3.71 | | 18496.41 |
| Q49ZZ3 | Uncharacterized protein | 0.48 | -0.43 | 2.19 | -1.94 | 0.098 | 0.207 | 12.11 | 7.57 | 14.10 | 1.07 | 0.66 | 1.93 | | 13089.52 |
| Q49VV4 | Uncharacterized protein | -0.85 | -0.21 | -6.07 | -1.49 | 0.001 | 0.344 | 26.88 | 34.85 | 22.67 | 2.36 | 3.03 | 3.10 | | 35163.36 |
| Q49VF9 | Uncharacterized protein | 0.19 | -0.24 | 0.78 | -0.98 | 0.549 | 0.527 | 30.95 | 23.10 | 26.16 | 2.72 | 2.01 | 3.58 | | 18322.37 |
| Y954 | Uncharacterized protein | -1.45 | -0.52 | -11.75 | -4.26 | ≤ 0.001 | 0.013 | 2.55 | 6.86 | 3.32 | 0.22 | 0.60 | 0.45 | | 13303.58 |
| UP355 | Uncharacterized protein | -0.11 | 1.03 | -0.34 | 3.34 | 0.813 | 0.038 | 2.43 | 2.80 | 1.06 | 0.21 | 0.24 | 0.15 | | 15415.07 |
| Y2125 | Uncharacterized protein | -0.54 | 0.35 | -2.12 | 1.38 | 0.105 | 0.384 | 3.84 | 5.72 | 2.84 | 0.34 | 0.50 | 0.39 | | 29339.25 |
| Q49W44 | Uncharacterized protein | -0.85 | -1.09 | -1.96 | -2.53 | 0.133 | 0.097 | 0.26 | 1.04 | ** | 0.02 | 0.09 | ** | | 13650.65 |
| Q49ZI5 | Uncharacterized protein | -0.29 | 0.00 | -1.21 | -0.01 | 0.339 | 1.000 | 0.99 | 1.41 | ** | 0.09 | 0.12 | ** | | 14050.32 |
| Y1625 | Uncharacterized protein | -0.78 | -0.37 | -4.52 | -2.14 | 0.006 | 0.157 | 0.65 | 1.45 | ** | 0.06 | 0.13 | ** | | 18964.24 |
| Y1806 | Uncharacterized protein | 0.30 | -0.78 | 1.11 | -2.93 | 0.372 | 0.064 | 1.42 | ** | 1.95 | 0.12 | ** | 0.27 | | 33174.24 |
| Q49VP3 | Uncharacterized protein | 1.41 | -0.52 | 2.35 | -0.87 | 0.080 | 0.554 | 4.71 | ** | 3.79 | 0.41 | ** | 0.52 | | 27936.33 |
| Q49YQ1 | Uncharacterized protein | -0.92 | 0.52 | -1.72 | 0.98 | 0.180 | 0.527 | ** | 2.54 | 0.58 | ** | 0.22 | 0.08 | | 22279.14 |
| Q49UH8 | Uncharacterized protein | 3.21 | 3.21 | 20.60 | 20.60 | ≤ 0.001 | ≤ 0.001 | 10.88 | ** | ** | 0.96 | ** | ** | | 73717.94 |
| Q49X01 | Uncharacterized protein | 2.47 | 2.47 | 21.00 | 21.00 | ≤ 0.001 | ≤ 0.001 | 5.69 | ** | ** | 0.50 | ** | ** | | 77515.47 |
| Q49XC4 | Uncharacterized protein | 3.83 | 3.83 | 38.03 | 38.03 | ≤ 0.001 | ≤ 0.001 | 16.04 | ** | ** | 1.41 | ** | ** | | 56058.72 |
| Y0419 | Uncharacterized oxidoreductase | -1.06 | 0.00 | -5.26 | 0.00 | 0.003 | 1.000 | ** | 2.29 | ** | ** | 0.20 | ** | | 24895.40 |
| Q4A067 | Uncharacterized protein | 0.00 | 1.49 | 0.00 | 5.40 | 1.000 | 0.005 | ** | ** | 0.35 | ** | ** | 0.05 | | 10999.22 |
| ^1^ Accession number provided by Uniprot Database (http://www.uniprot.org/). | | | | | | | | | | | | | | | |
| ^2^ Obtained from limma’s topTable by subtracting the average expression in log2 scale against that of ATCC 15305 strain. | | | | | | | | | | | | | |  |  |
| ^3^ Estimate of the Student’s t statistic comparing expression against that of the ATCC 15305 strain. | | | | | | | | | | | | | |  |  |
| ^4^ p-value from the Student’s t distribution. Proteins with p-value ≤ 0.05 were considered regulated among the strains. | | | | | | | | | | | | | |  |  |
| ^5^ Amount (in ngram) of this protein species identified by proteomic analysis. | | | | | | | | | | | | | |  |  |
| ^6^ Percentage of this protein species, compared to the total number of proteins identified in this strain. | | | | | | | | | | | | | |  |  |
| ** Protein not detected in this strain | | | | | | | | | | | | | |  |  |
